# Interactions of Multiple Biological Fields in Stored Grain Ecosystems

**DOI:** 10.1038/s41598-020-66130-6

**Published:** 2020-06-09

**Authors:** Z. D. Wu, Q. Zhang, J. Yin, X. M. Wang, Z. J. Zhang, W. F. Wu, F. J. Li

**Affiliations:** 1 Jilin University, Changchun, China; 2 Academy of National Food and Strategic Reservation Administration, Beijing, China; 3 University of Manitoba, Winnipeg, Manitoba Canada

**Keywords:** Biophysics, Ecology

## Abstract

Biological entities such as fungi in stored grain evolve and interact with the environment in similar fashions as physical fields. An experiment was conducted to study the behavior of the biological field of fungi in stored grain, as well as the interactions between the biological field of fungi and the physical fields of temperature and moisture. A framework of the biological field is presented to describe biological systems in which multiple biological entities co-exist and interact among themselves and with the surrounding environment. The proposed biological field describes the spatio-temporal distribution of a biological entity and its ability of influencing (or being influenced by) the surrounding biotic and abiotic entities through exchange of energy, matter, and/or information. The strength of a biological field of fungi was quantified as the rate of energy conversion by fungi from grain starch to heat. The experimental data showed that the strength of biological field of fungi in stored grain varied in both space and time, with the maximum field strength of 120–133 *W m*^*−3*^ occurred at the location where the biological field of fungi interacted strongly with the temperature and moisture fields.

## Introduction

A biological system is a complex network of biologically relevant entities that interact with each other and with the surrounding environment. The behaviour of each entity varies spatially and temporally. Taking stored grain as an example, Dunkel^1^ described the stored grain as an ecosystem that was a combination of interacting biotic communities which in turn interact with their abiotic environment. The biotic communities include grain, fungi, insects, mites, birds, rodents, or even human beings, while the abiotic environment includes storage structures (silos, bins, bags, etc.), temperature, humidity, and gases. Each biological entity may produce a set of outputs (energy, matter, and/or information) from a given set of inputs at a certain state (time). Researchers have attempted to develop quantitative models to characterize biological systems in terms of their variation and evolution in space and time. For example, the Eden^2^ model has been used for predicting spatial patterns in bacterial colonies. The diffusion-type differential equations have been developed to describe the time evolution of spatial fluctuations in biological systems^3,4^. A limitation of studies reported in the literature is the lack of vigorous consideration of interactions among the co-existing biological entities and between the biotic and abiotic entities. For example, in a stored grain system, the abiotic factors such as temperature and moisture dictate the germination and development of fungi, while the growth of fungi produces heat and moisture to change the temperature and moisture, which in turn accelerates the fungal growth and may also lead to the emergence of other biological entities (such as different fungal species or bacteria) that favor different temperatures or moistures. The upper temperature limit for eukaryotic organisms is about 60 °C^5^; therefore, heat produced by fungi may also lead to temperature rises sufficiently high to cause the thermal death of fungi. Describing this complex network of biologically relevant entities, and interactions among themselves and with the physical environment is extremely challenging. A well-established concept in physics, the **field**^6^, may provide a powerful tool to study the intertwined complex biological systems.

A field is generally defined as a quantity that vary continuously in space and time. The quantity can be represented by a number (scalar field, e.g., temperature) or a vector or tensor (vector or tensor field, e.g., force and stress). The field theory has been used successfully to explain and predict many physical phenomena. Attempts have also been made to use the field concept to describe and predict biological systems. Torday^7^ stated that “biologic organisms act like fields”. It was further explained that “pleiotropy forms the basis for a dynamic way of understanding the generation of biology from physics due to their mutual origins in the Big Bang”. A significant example of using the field concept in biological systems is Kurt Lewin’s field theory in psychology^8^. Lewin’s field theory was based on the argument that the order of co-existing facts in a psychological or social situation could be viewed as a life space (field), and it is possible to understand and predict the behaviour of individuals and groups by constructing a life space comprising the psychological forces influencing their behaviour^8^.

While the field concept has a great potential in describing the complex biological systems, there are no commonly accepted definitions of biological field. It is proposed in the current study that a biological field is the spatio-temporal distribution of a biological entity, with abilities of influencing (interacting with) the surrounding biotic and abiotic entities through exchange of energy, matter, and/or information*.* In the context of stored grain ecosystems, a biological field of fungi has an ability of influencing the temperature field in the grain by converting grain starch to heat^9^. Another example is *Rhizobium*–legume symbiosis where the biological field (spatial and temporal distribution) of *Rhizobium* involves the exchange of molecular signals (information) between the bacteria and the plant host^10^. A challenge in applying the field concept to biological systems is how to quantify the field strength. The objective of this research was to study the behavior of biological fields and develop a framework of biological field theory to quantify the biological systems, with an emphasis on interactions among the co-existing biotic and abiotic fields. Fungi in stored grain ecosystems were used as an example to illustrate and verify the proposed framework of biological field theory.

## Materials and Methods

### Experimental set-up and procedure

Germination and growth of fungi in stored grain occur in a complicated manner involving many biological, physical and chemical variables and conditions. In this experiment, two most significant physical variables associated with fungal growth in stored grain – temperature and moisture, were manipulated to study the behavior of biological fields of fungi and their interactions with physical fields. To mimic the conditions of grain spoilage by fungal growth in actual storage facilities, the grain was not inoculated and the naturally existing fungi in the grain were expected to germinate under favorable combinations of grain temperature and moisture.

#### Test bin and measurements of physical and biological fields

**Figure 1:**
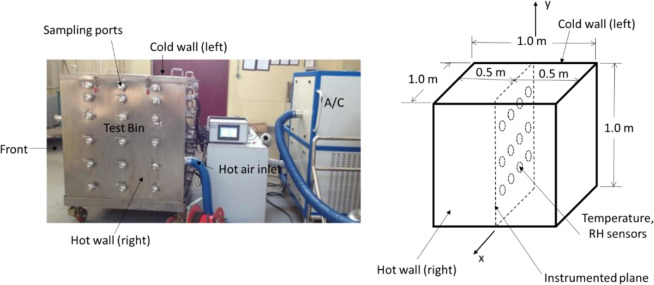
Schematic diagram of test bin for conducting experiment.

A small grain storage bin of 1 m × 1 m × 1 m was designed and constructed with stainless steel to conduct the experiment (Fig. 1). To mimic large scale grain storage systems, the walls of the test bin were thermally insulated to simulate the conditions of heat flow in a large grain bulk which has relatively low thermal conductivity. In a typical grain storage facility, the walls of containing structure are subject to temperature changes due to changing outdoor conditions, which would result in temperature gradients within the grain bulk. These temperature gradients are often the cause of fungal growth in the stored grain. To simulate this condition in the test bin, the temperatures of the two side (left and right) walls were controlled by circulating conditioned air through them to create a temperature gradient across the grain bulk. The left (cold) wall was controlled at 10 °C and the right (hot) wall at 44 °C. In other words, a temperature field increasing from 10 °C to 44 °C was established in the grain bulk. It was expected that along this temperature gradient (between the left and the right walls), there existed a point (or region) where temperature would be suitable for fungi to germinate at the given grain moisture. In other words, the biological (fungi) and physical (temperature) fields would interact at this point (region).

Temperature and RH sensors (Model FCS-1, Ningxia Dongda Hengfeng Science & Technology Company Ltd., Ningxia, China) were placed along a measurement plane (x-y plane in Fig. 1) in the middle between the front and back walls. The sensors were spaced at 0.1 m in both the x- (lateral) and the y- (vertical) directions to continuously record the temperature and RH distributions in the grain mass (Fig. 1).

#### Test grain

The grain used in the test was wheat grown in a farm in Henan Province, China. Following the ASABE Standards^11^, the initial moisture content of wheat was measured to be 14.4% wet basis *(w.b*.). The bulk density of wheat was determined to be 786.20 *kg m*^*−3*^ using the method recommend by NSPRC^12^. The thermal conductivity for the test wheat was reported by Yin^13^ to be 1.98 *W m*^*−*1^ *°C*^*−*1^ and the specific heat capacity 1.78 *kJ kg*^*−1*^ *°C*^*−1*^, which increased to 2.84 *kJ kg*^*−1*^ *°C*^*−1*^ after the wheat was naturally infected (spoiled) by fungi.

Following the China National Standard^14^ for identifying fungi in stored grain, naturally existing fungi before experiment were identified to be mostly *Aspergillus glaucus*, with some *Aspergillus flavus and Aspergillus candidus*. Different methods have been used to assess fungal invasion in stored grains, such as spore counts^15^ and ergosterol concentrations^16,17^. For spore counts, the procedure outlined in the SAG Standards^15^ was followed. Specifically, 10 g of wheat was mixed with 30 mL of distilled water in a test tube, which was then shaken for 1 min to strip fungal spores from the wheat. The liquid was then filtered through a 300 mesh cloth. The filtrate was placed on a hemocytometer and observed under a microscope to count the number of fungal spores. The initial fungal spore count was determined to be 6 × 10^5^ g^−1^, which is considered to be normal for unspoiled grain^15^. Wheat samples were also sent to a commercial laboratory (Beijing Biotech Pack, Beijing, China) for determination of ergosterol. The measured concentration of ergosterol was 10.02 μg g^−1^.

During the experiment, a grain sampling probe was used to take 20 g of wheat sample at each measurement point approximately every 15 days for fungal measurements. The stainless steel sampling probe was manually pushed into the grain bulk through the sampling ports on the front face of the test bin to take samples (Fig. 1).

### Quantification of field strength of biological fields

A fundamental concept in biology is energy cycling within the system and exchange with the environment. Specifically, all cells use energy from their environment to grow, make new parts, metabolize, and reproduce. In a stored grain ecosystem, dormant seeds serve as an energy source and as a habitat for other biological entities, including fungi, bacteria, insects, and mites^1^. Therefore, it is proposed herein to use the rate of energy utilization/conversion by the biological entity to define the field strength of a biological field. Specifically, the biological field strength is defined as the ability of biological entities to utilize, convert, or dissipate energy. Accordingly, the unit of biological field strength is expressed as *J s*^*−1*^ *m*^*−3*^ or *W m*^*−3*^, representing the rate of energy utilization/conversion/dissipation in a specific space (*m*^3^). A general mathematical expression can be used to define the strength of a biological field as follows:

1 $$P(x,y,z,t)=\frac{dQ(x,y,z,t)}{dt}$$

where *P* is the field strength (*W m*^*−3*^*), Q* is the energy utilized, converted or dissipated in a specific space that is influenced by the biological field at a given time (*J m*^*−3*^), *t* is time (*s*), and *x, y*, and *z* are space coordinates (*m*).

Energy *Q* in a biological system (field) may exist in various forms, including heat *Q*_*H*_; mechanical energy *Q*_*M*_ (e.g., phase change, aggregation and dispersion); chemical energy *Q*_*E*_ stored in molecular bonds (e.g., starch, fat, protein, cellulose); energy for biological growth and reproduction *Q*_*B*_; and energy for producing or decomposing organic substances and other biological entities (e.g., organic acids, alkaloids, biopheromones, and biological toxins) *Q*_*R*_. Therefore, the total energy term in Eq. 1 can be generally expressed as:

2 $$Q={Q}_{H}+{Q}_{M}+{Q}_{E}+{Q}_{B}+{Q}_{R}+\cdots $$

In this study, the measured temperature data was used to calculate the rate of energy conversion as heat production by fungi in stored grain. While most heat produced by fungi was absorbed by grain, causing the grain temperature to rise, some heat was lost due to conduction and convection, and some was spent on converting liquid water to vapor. Therefore, the total heat production by fungi could be determined as follows:

3 $$Q={Q}_{H}={E}_{T}+({E}_{c}+{E}_{v}+{E}_{E})$$

where *E*_*T*_ is the sensible heat absorbed by grain (raising the grain temperature) in a unit space (*J m*^*−3*^), *E*_*C*_ is the conduction heat loss from a unit space (volume) to the surrounding grain mass (*J m*^*−3*^), *E*_*V*_ is the convection heat loss from a unit space to the surrounding grain mass (*J m*^*−3*^), and *E*_*E*_ is the heat of evaporation (sensible heat consumed for converting moisture to water vapor) in a unit space (*J m*^*−3*^).

## Results

### Temperature and fungal fields in test bin

**Figure 2:**
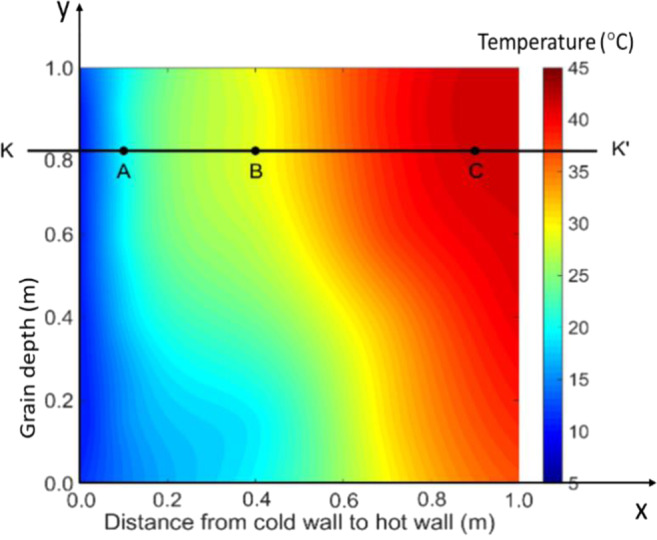
Temperature field (distribution) in the test bin on day 12.

At the start of experiment, the grain temperature increased gradually due to conductive heat transfer between the grain and the (cold and hot) walls. On day 12, the grain temperature reached a stable (equilibrium) condition, increasing from approximately 10 °C at the left wall to 44 °C at the right wall in the upper part of the bin (Figs. 2 and 4). There was a clear temperature gradient in the x-direction (from the cold to the hot wall) at any given grain depth. Some variations in grain temperature were also observed in the y-direction (along the grain depth) due to the effect of cold air inlet (lower left corner) and hot air inlet (upper right corner), causing higher temperature in the upper portion of the bin, in particular along the hot wall (Fig. 2). The overall observation indicated that the temperature distribution (field) in the stored grain was physically driven (mainly through conduction) by the temperature difference between the cold and hot walls at this stage.

**Figure 3:**
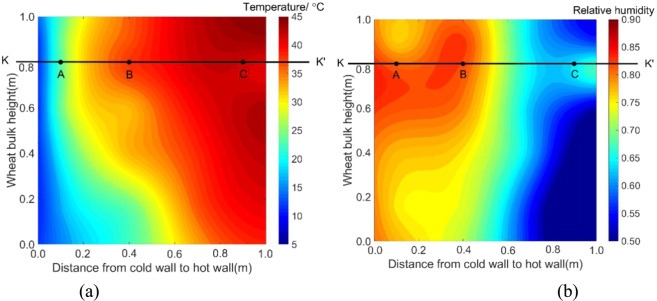
The measured temperature and RH fields of mid-plane in test bin on day 36. (**a**) temperature, (**b**) RH (Note: a sampling port was near C, which caused a slight temperature drop and RH rise).

**Figure 4:**
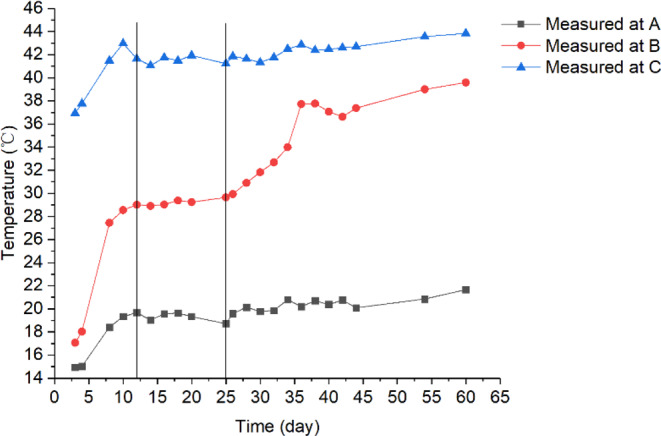
Variations of measured temperature during the experimental period at three locations A(0.1, 0.8), B(0.4, 0.8) and C(0.9, 0.8) in the test bin.

**Figure 5:**
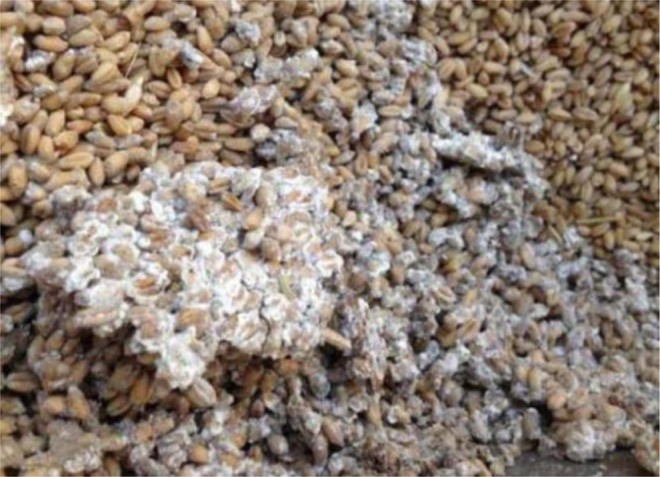
Spoiled (mouldy) grain found at location B (0.8 m from the bin bottom and 0.4 m from the cold wall) on day 60.

On day 36, a clearly noticeable temperature rise occurred at a point located 0.8 m from the bin bottom and 0.4 m from the cold wall (this location is denoted as **B**, with coordinates *x* = 0.4 and *y* = 0.8) (Fig. 3a). To further examine the temperature changes at location **B**, the time history of temperature was plotted and compared with other two locations A and C, both were at the same grain depth as **B** (Figs. 3 and 4). It could be seen: (1) temperature at **B** started to increase on day 25, and reached a peak of 38 °C on day 38; and (2) little temperature increase (besides some fluctuations) was observed at locations **A** and **C** (Fig. 4). At the same time, high moisture (measured RH) was observed at **B** (Fig. 3b). These observations indicated that a biological field of fungi was emerging at **B**, causing temperature and humidity to rise. The grain samples taken on day 36 confirmed that the fungal spore count at **B** had increased to 8 × 10^6^ g^−1^, which is more than 10 times the initial value (at the start of experiment). The grain samples also showed an increased ergosterol concentration of 43.52 μg g^−1^, which is 4 times the initial value. Both the spore count and ergosterol concentration indicated significant fungal growth at **B** on day 36. The fungal spore count increased further to 8 × 10^7^ g^−1^ and the ergosterol concentration to 50.06 μg g^−1^ on day 54 (the last grain sampling day). When the test bin was opened at the end of experiment (day 60), moldy grain was found in a small region round **B** (Fig. 5). Following the method of NSRPC^14^, *A. flavus* was identified as the main species, with some *A. candidus*, in the moldy grain.

### Interactions of fungal field with temperature and moisture fields

**Figure 6:**
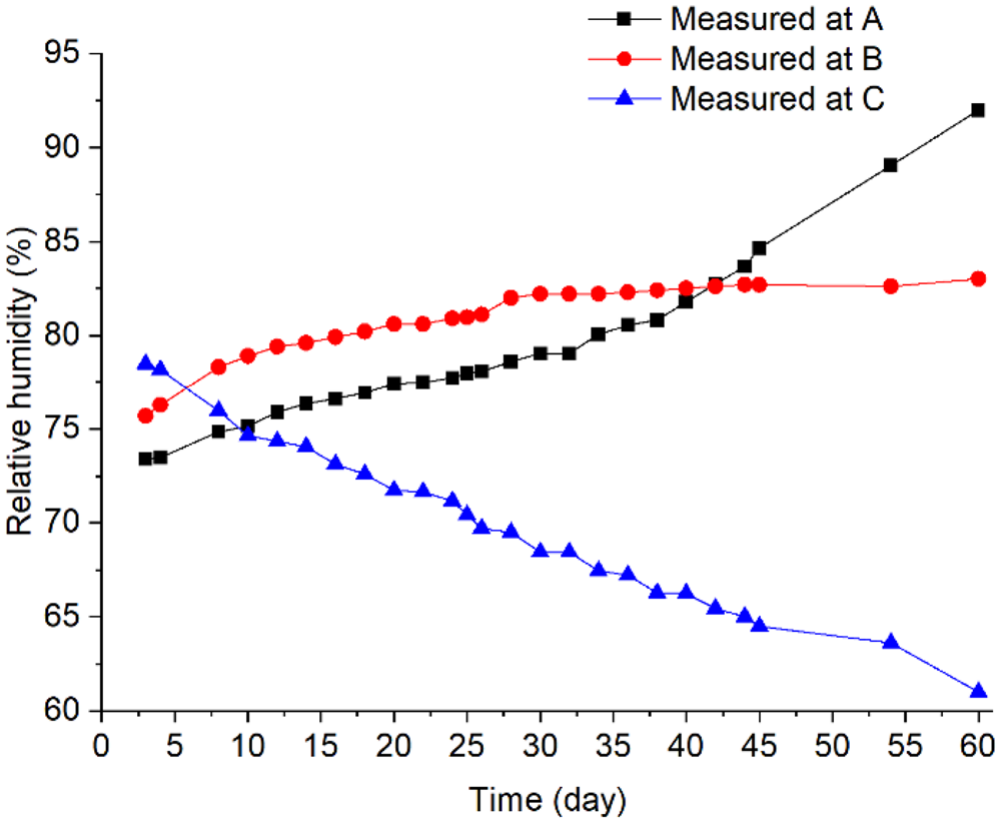
The measured relative humidity at locations (**A**–**C**).

To explore the multi-field interactions, the measured temperature and RH at **B** were compared with the two neighboring locations **A** (0.1, 0.8) and **C** (0.9, 0.8). Physically, points **A** and **C** located at the same grain depth as **B** (0.8 m from the bin bottom), but **A** was closer to the cold wall (0.1 m) and **C** closer to the hot wall (0.1 m). The measurements showed that temperature and RH at **B** were 29 °C and 80% on day 12 (after reaching equilibrium) (Figs. 4 and 6), respectively, which are suitable for fungi to germinate (the minimum condition of temperature and RH for the growth of main grain storage fungi: >20 °C and >65%RH)^18^. In comparison, temperature at **A** was 19 °C, which is lower than the minimum temperature for fungal germination although the relative humidity (83%) at **A** was suitable for fungi (Figs. 4 and 6). At point **C**, temperature was 42 °C, which is suitable for fungal germination, but the relative humidity decreased from 74% RH to 60% RH very quickly, because grain was dried near the hot wall (Figs. 4 and 6), which is not suitable for fungal germination. This comparison of environmental conditions among the three points showed that a suitable state of physical fields (both temperature and RH) was reached at **B** first, and strong interaction occurred between the biological and physical fields at this point: fungi germinated, grew and sporulated, and temperature rose significantly due to heat production by fungi. Whereas, the suitable state of physical fields was not reached at points **A** and **C** and no interaction was observed.

### Strength of biological field of fungi

**Figure 7:**
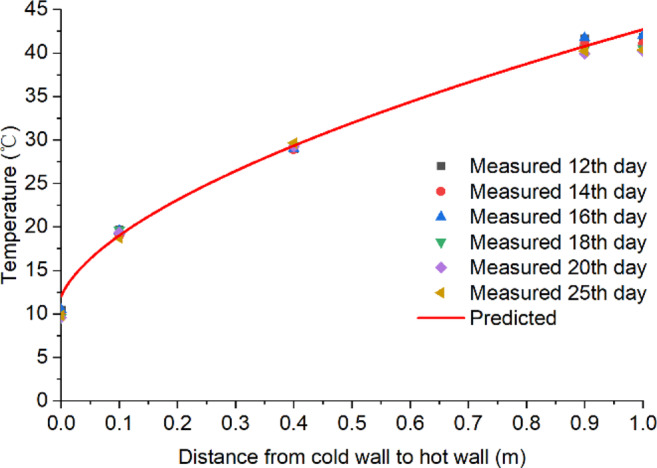
Temperature profile between the cold and hot walls after reaching equilibrium but before fungal emergence (from days 12 to 25) at grain depth of 0.8 m from bottom (covering locations **A–C**).

To determine the strength of fungal field in the stored grain, the measured temperature data was used in Eq. 3 to calculate heat production by fungi. First, the sensible heat absorbed by grain (*E*_*T*_) was calculated from the grain temperature rise *ΔT*. Since the left and right walls were maintained at constant temperatures of 10 °C and 44 °C, respectively, grain temperature would increase linearly from 10 °C at the left wall to 44 °C at the right wall once the steady-state was reached. However, the grain was dried more near the hot wall, resulting in a lower thermal conductivity *k* value and this decrease in thermal conductivity from the cold to the hot wall resulted in a slightly curved temperature profile before fungus emergence (Fig. 7). The measured temperature (Fig. 4) indicated that the equilibrium condition was reached on day 12 and lasted until day 25 when the temperature started to rise due to fungal heating. Using the temperature data for days 12, 14, 16, 18, 20 and 25 to represent the temperature profile before fungal heating, regression analysis was performed using the Origin Data Analysis and Graphing Software (OriginLab Corporation, Northampton, MA, USA) to generate an equation to mathematically predict the temperature profile between the cold and hot walls before fungal emergence (Fig. 7):

4 $${T}_{B}={10.5}+{32.1}{(x+{0.006})}^{0.6}\,{R}^{2}={0.999}$$

where *T*_*B*_ is the grain temperature profile (°C) between the cold and hot walls before fungus emergence and *x* is the distance from the cold wall (m).

**Figure 8:**
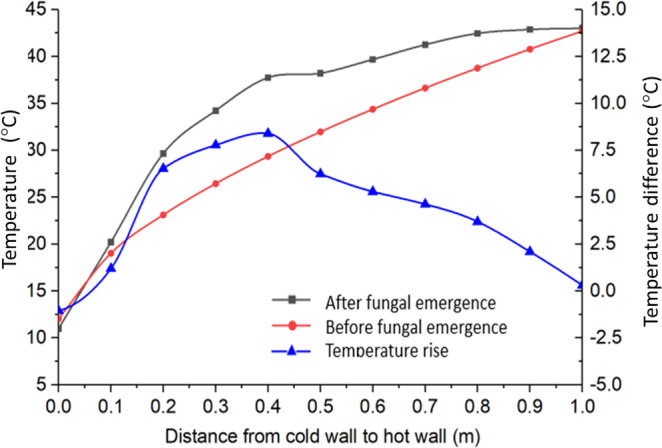
Measured temperature profile with fungal heating (after fungal emergence) and temperature profile before fungal emergence (predicted by Eq. 4), for day 36, and the corresponding temperature rise due to fungal heating.

When fungi started to grow at **B** on day 25, heat produced by fungi caused the grain temperature to rise around **B**, causing the temperature profile to rise above that at the equilibrium, and the grain temperature rise at a given time and location between the cold and the warm walls could be calculated as the difference between the two temperature profile curves (Fig. 8). Generally speaking, the temperature rise *ΔT* would be a function of both time (t) and location (x, y, z). Mathematically, the temperature rise due to heat production by fungi could be calculated from the temperature difference before and after fungus emergence as follows:

5 $$\Delta T(x,y,z,t)={T}_{A}(x,y,z,t)-{T}_{A}(x,y,z)$$

where *ΔT* is the temperature rise due to fungal activities at a given time and location (°C) and *T*_*A*_ is the measured grain temperature at a given time after fungal emergence (°C). Note: only the x-direction was considered in this experiment. Based on the temperature rise calculated by Eq. 5, heat absorbed by grain was calculated as follows:

6 $${E}_{T}=\frac{S\rho \Delta V\Delta T(x,y,z,t)}{\Delta V}=S\rho \Delta T(x,y,z,t)$$

where *S* is the specific heat of grain (*J kg*^*−1*^ *°C*^*−1*^), *ρ* is the density of grain (*kg m*^*−3*^), *ΔV* is the space (volume of grain) influenced by the fungal activities (*m*^3^).

The conduction heat loss (*E*_*C*_) was determined by considering heat transfer from the center of a small space of *Δx* = 0.1 *m*, *Δy* = 0.1 *m*, and *Δz* = 0.1 *m* (*ΔV* = *ΔxΔyΔz* = 0.001 *m*^3^) to the surrounding grain mass. The size of this small space was based on the spacing of temperature and RH sensors in the test bin (0.1 *m* by 0.1 *m*). Fourier’s law was used to calculate *E*_*C*_ as the sum of heat loses by conduction from all six faces of the cubical space (*ΔxΔyΔz*) under consideration:

7 $${E}_{C}=\frac{k\Delta t}{\Delta x\Delta y\Delta z}\mathop{\sum }\limits_{i=1}^{6}\frac{{A}_{i}\Delta {T}_{i}}{{l}_{i}}$$

where *k* is the thermal conductivity of grain, *Δt* is the time increment (*s*), *A*_*i*_ is the area of one of the six faces of the cubical space (*m*^2^), e.g., *A*_*1*_ = *ΔyΔz* for the face in the positive *x*-direction, *ΔT*_*i*_ is the measured temperature difference between the center of the cubical space (*ΔxΔyΔz*) and the corresponding face *A*_*i*_ at a given time (*°C*), and *l*_*i*_ is the distance from the center of the cubical space and the corresponding face A_i_ (m), e.g., *l*_*1*_ = *0.5Δx* for the face in the positive *x*-direction.

Heat loss by convection is due to the movement of air through the interstitial spaces in the grain bulk. Given that the grain was not aerated in the experiment and bin was sealed (minimal free convection), there was little air movement, and therefore, heat loss by convection *E*_*V*_ was negligible. Similarly, heat of evaporation was also negligible because the grain moisture changed little during experiment. Therefore, Eq. 3 was reduced to:

8 $${Q}_{H}={E}_{T}+{E}_{C}$$

Substituting Eqs. 6 and 7 into Eq. 8, and then into to the definition of field strength given by Eq. 1, the strength of the biological field of fungi in stored wheat was determined as follows:

9 $$P=\frac{\varDelta {Q}_{H}}{\varDelta t}=\frac{{E}_{T}}{\varDelta t}+\frac{{E}_{C}}{\varDelta t}=\frac{S\rho \varDelta T(x,y,z,t)}{\varDelta t}+\frac{k}{\varDelta x\varDelta y\varDelta z}\mathop{\sum }\limits_{i=1}^{6}\frac{{A}_{i}\varDelta {T}_{i}}{{l}_{i}}$$

**Figure 9:**
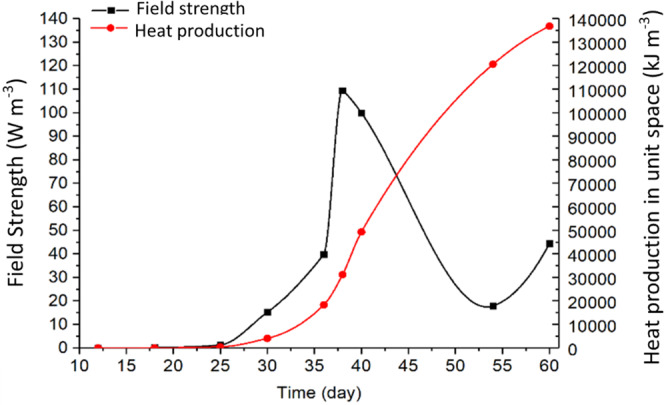
Variation of heat production and field strength of fungi in a small space surrounding B (0.4, 0.8, 0) in the grain stored in a test bin of *1 × 1 × 1 m*.

**Figure 10:**
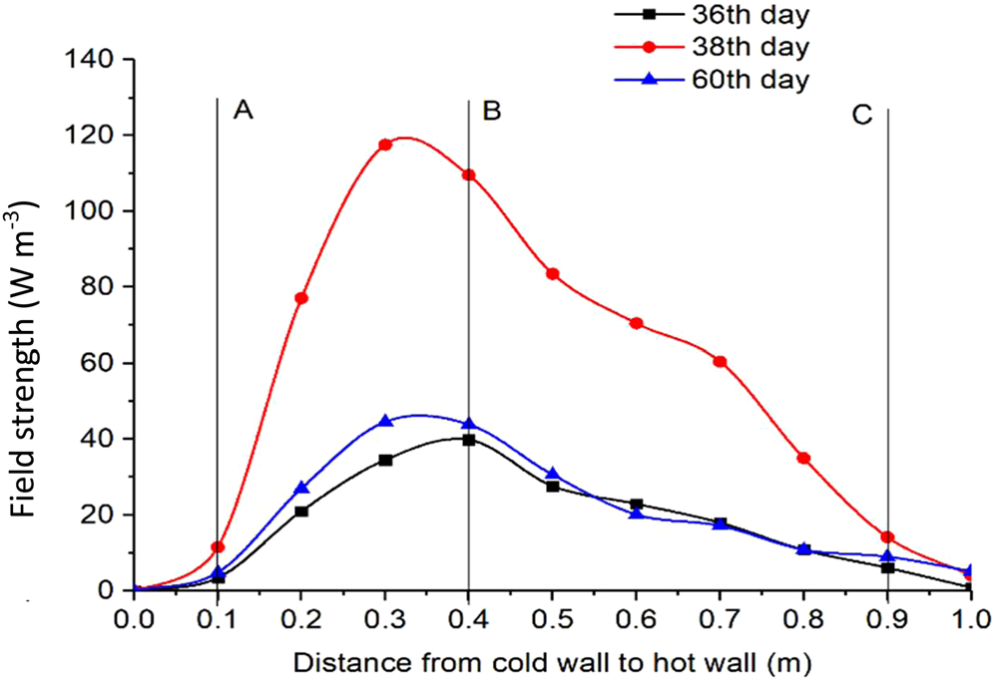
Variation of field strength from the cold to hot walls at a grain depth of 0.8 m in the test bin on different days.

Using the measured grain temperature *T*_*A*_ in Eq. 5 and *T*_*B*_ from Eq. 4 to calculate *ΔT*, and the field strength *P* was calculated by Eq. 8 as a function of time *t* (Fig. 9) for location **B**, and as a function of location *x* on days 36, 38 and 60 (Fig. 10). A time step *Δt* = 86400 *s* (1 day) was used in the calculation to ensure that temperature rise was sufficiently high to be accurately measured by the temperature and RH sensors between time steps.

The biological field of fungi remained “latent” until day 25 (i.e., the filed strength increased little between day 5 and day 25, Fig. 9). From day 25 to 38, the biological field developed rapidly (Fig. 9). The strongest field strength was measured to 110 *W m*^*−3*^ at **B** on day 38. And the heat production accumulated in the small space (0.1 × 0.1 × 0.1 m) surrounding **B** from day 25 to day 38 was about 30 *MJ m*^*−3*^ (Fig. 9). Using the strongest field strength at **B** (110 *W m*^*−3*^) on day 38, the heat production was calculated to be 9.5 *MJ m*^*−3*^ *d*^*−1*^. Based on the cellular respiration equation under the aerobic condition (C_6_H_12_O_6_ + O_2_ → CO_2_ + H_2_O + heat), This amount of heat production was estimated to be equivalent to the consumption of 3.4 *mol m*^*−3*^ *d*^*−1*^ (604 *g m*^*−3*^ *d*^*−1*^) of glucose, production of 20.4 *mol m*^*−3*^ *d*^*−1*^ (367 *g m*^*−3*^ *d*^*−1*^) of H_2_O, or 898 *g m*^*−3*^ *d*^*−1*^ of CO_2_. Assuming that wheat contained 75% starch (commonly 70% to 80%), the dry matter consumption by fungi was estimated to be about 823 *g m*^*−3*^ *d*^*−1*^ at the peak of field strength.

The field strength started to decrease after day 38, while heat continued to accumulate (Fig. 9). It is interesting to note that the field strength started to increase again after reaching a low point (about 18 *W m*^*−3*^) on day 54, indicating the possibility of emergence of another biological field. This is a typical phenomenon of succession of microorganisms in stored grain ecosystems – rising temperature and moisture, or changes in levels of various gases (e.g., oxygen and carbon dioxide) level created a favorable environment for another biological entity to grow.

The spatio-temporal distribution of field strength is illustrated in Fig. 10. On day 36, the field strength peaked at **B**. While the shape of the curve remained about the same, the field strength reached the highest level on day 38, with the peak (about 120 *W m*^*−3*^) slightly shifted to the left from **B**. The field strength started to decrease after day 38 because of excessive heat production, causing temperature to rise beyond the optimal range for fungi, or exhaustion of oxygen and nutrients, but the spatial distribution (the curve shape) of field strength remained the same (Fig. 10). The above observations clearly indicated that the distribution and evolution of biological entities (fungi) measured in this experiment behaved like a physical field in that the field strength varied spatio-temporally.

## Discussion

### Multi-field interactions/coupling

**Figure 11:**
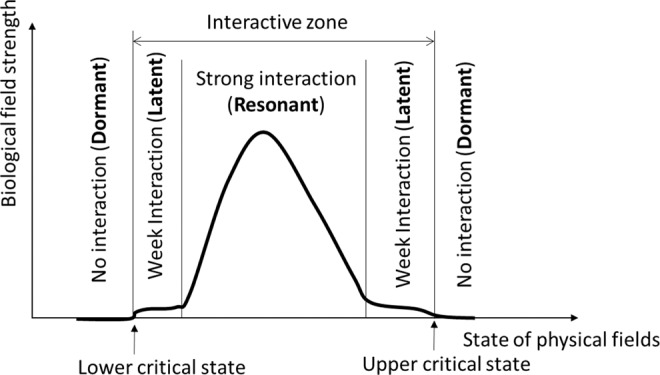
Schematic illustration of different interaction states between biological and physical fields.

Examining the field strength of fungi in Fig. 9 along with the measured temperature (Fig. 4) and humidity (Fig. 6) reveals the general characteristics of interaction between biological and physical fields. A biological field (fungus in this case) stays ***dormant*** (with a field strength near 0) when it is not interacting with the physical fields (Fig. 11). When the environmental conditions (the state of co-existing physical fields) become favorable to the biological entity, the biological field starts to develop (grow and reproduce) with time and in space, and consequently the biological field strength starts to increase. We shall define this state of physical fields (environmental conditions) that triggers the changes in the biological field as the **critical state** and the region within the critical state as the interactive zone (Fig. 11). Based on the rate of change in field strength, the interaction can be categorized into three states: the dormant state (no interaction), the latent state (week interaction), and the resonant state (strong interaction) (Fig. 11). In the dormant state, the environment conditions are not suitable for the biological entity to grow and reproduce, or even to survive. Under such conditions, the biological entity remains dormant (or no longer alive) and the field strength is close to or at zero. In the latent state, the basal metabolism of biological entity is maintained (latent development and accumulation). In the resonant state, the environmental conditions are optimal for the biological entity to grow and reproduce; and also energy/matter exchange between the biological entity and the environment may change the environment to a more favorable state for the biological entity. This positive feedback phenomenon (self-stimulated growth) is analogous to resonance in physics.

In the context of fungi in stored grain, a biological field of a fungal species co-exists with other biological fields (other fungal species, insects, bacteria, etc.) and with several physical fields, including temperature, moisture, and gas (CO_2_, O_2_) fields. Fungi in the stored grain are dormant at low temperature (i.e., <7 °C) (the dormant state below the lower critical state, Fig. 11) and starts to germinate when temperature becomes favorable (assuming a suitable grain moisture exists), reaching the lower critical state and causing the interactions. Slightly above the lower critical state is the week interaction state in which the temperature is suitable for fungi to maintain their basic life processes, and a small amount of heat of respiration is produced. In this latent state, the field strength is measurable but very low. When the temperature becomes optimal for fungi, they will grow rapidly. For example, the optimal growth temperature for a typical stored grain fungi *A. flavus* is 40–45 °C^18^. The growth of fungi produces heat and water through respiration, causing the temperature and moisture to rise, which in turn will accelerate the fungal growth. This positive feedback (the resonant state) leads to strong interaction and sharp increases in field strength (Fig. 11). As fungi continue to grow, energy and matter exchanges with the environment will further modify the physical fields. Some possible outcomes are: less grain (energy supply) available (as it has been consumed by fungi); higher temperature and moisture, lower oxygen level, and higher carbon dioxide level, or accumulation of other metabolites. One of the above changes of physical conditions will limit the fungal growth as time progresses. For example, as more heat is produced by fungi, temperature will become too high for fungal growth (e.g., 45 °C for *A. flavus*). Consequently, the field strength starts to decrease, and eventually approach zero when all fungi are killed off after temperature reaches the thermal death limit for fungi (about 60 °C). This will cause the biological field to return to a dormant state (or be completely destroyed), and thus interactions stop (beyond the upper critical state, Fig. 11).

**Figure 12:**
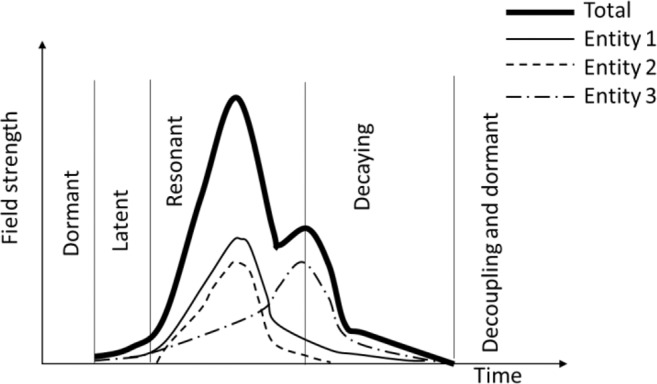
Schematic illustration of different stages of interaction between multiple biological fields in the time domain.

While Fig. 11 describes the multiple field interactions as a function of physical variables (e.g., temperature and moisture), we may also describe the field interactions in the time domain for co-existing fields (Fig. 12). When multiple biological fields co-exist (e.g., biological entities 1, 2 and 3 in Fig. 12), the total field strength reflects the combined ability of energy conversion by all biological entities involved. Each individual biological field (entity) has its own critical state (optimal environmental conditions) and the interactive zones are different for individual co-existing fields. For example, the optimal temperature and relative humidity for two typical fungi in stored grain, *A. flavus* and *A. candidus*, are 40–45 °C and 45–50 °C, respectively; and relative humidity 80–85% and 75–80%, respectively^18^. When the environmental conditions are not suitable for any biological entities in the system, the overall biological field stays dormant (Fig. 12). The total field enters the latent state when the environmental conditions bring one or more biological entities out of dormancy. Resonance (self-stimulated growth) occurs when the environmental conditions become optimal for one or more biological entities in the system. The resonant condition reflects the interactions among the biological fields themselves, as well as with physical fields. For example, researchers have observed that fungi usually accompany or follow insect infestation in stored grain ecosystems^19^. Specifically, insects can start to grow at relatively low temperature and moisture, and their growth produces heat and water to increase temperature and water activity (moisture) to levels suitable for fungi to germinate^1^. This example shows that emergence of a biological field (insects) may interact with some physical fields (temperature and moisture), which in turn lead to the emergence of another biological field (fungi). This process may repeat, and more biological fields will emerge, resulting in a rapid increase in the overall field strength. As time progresses, the environmental conditions are altered by the growth of biological entities to become unfavorable to the biological entities. For example, metabolic heat production by insects and fungi may raise temperature to the level that is too high for the biological entities to grow or even survive, and consequently the biological fields start decaying, and eventually all interactions cease; the biological fields are considered to be decoupled from the physical fields and become dormant (Fig. 12).

### Quantifying strength of biological fields

The experimental results presented in this study demonstrated that biological fields are measurable through physical variables. Specifically, the proposed definition of field strength (Eqs. 1 and 2) for a biological field of fungi could be quantified using the heat of respiration by fungi, which was calculated from the measured temperature rise due to fungal activities. In essence, the temperature rise (*ΔT*) was used as an indicative state variable (ISV) in quantifying biological fields of fungi. Following this reasoning, we propose that for any biological fields, its field strength may be determined from the changes of ISV’s when using Eq. 2 to calculate energy components. This procedure can be mathematically described as follows:

10 $${Q}_{H}={f}_{H}(\Delta T)(x,y,z,t)\,{\rm{is}}\,{\rm{generalized}}\,{\rm{to}}\,{Q}_{i}={f}_{i}(\Delta {\lambda }_{i})(x,y,z,t)$$

where *Q*_*i*_ represents an energy component in Eq. 2, i.e., *Q*_*i*_ = *Q*_*H*_*, Q*_*M*_*, Q*_*E*_*, Q*_*B*,_ *or Q*_*R*_*, λ* is an ISV that reflects changes in the strength of a biological field (such as temperature), and Δ*λ* is the increment of ISV. For instance, *Δλ* could be the displacement increment for mechanical energy *Q*_*M*_, or the change in substance’s composition/concentration for chemical energy Q_E_, or the change in population density for *Q*_*B*,_ or the change in the gradient of gas concentration for *Q*_*R*_. Once the increments of ISV’s (Δ*λ*_*i*_) are determined (measured) for a biological system, the field strength can be quantified by using Eqs. 1 and 2.

## Conclusions

The concept of field has been successfully used in physics to describe and predict various physical phenomena. In this study, a framework is presented to use the field concept to describe the behaviour of biological systems in both space and time domains, and the interactions between the biological and physical fields. Specifically, the spatio-temporal distribution of a biological entity in a biological field can be quantified by its field strength, which is defined as the ability of the biological entity in influencing other physical and biological entities through exchange of energy. The laboratory experiment conducted in this study on fungal growth in stored grain confirmed the existence of biological fields of fungi in stored grain ecosystems. The field strength of a biological field could be calculated from the exchange of energy by considering changes of an indicative state variable(s) of the biological field, such as temperature.
