# Author Correction: Interactions of multiple biological fields in stored grain ecosystems

**DOI:** 10.1038/s41598-024-54618-4

**Published:** 2024-02-22

**Authors:** Z. D. Wu, Q. Zhang, J. Yin, X. M. Wang, Z. J. Zhang, W. F. Wu, F. J. Li

**Affiliations:** 1 Jilin University, Changchun, China; 2 Academy of National Food and Strategic Reservation Administration, Beijing, China; 3 University of Manitoba, Winnipeg, Manitoba Canada

Correction to: *Scientific Reports* 10.1038/s41598-020-66130-6, published online 09 June 2020

The original version of this Article contained an error in Equation 9.

In the original version of this article, under the Results section, where

$$ P = \frac{{\Delta Q_{H} }}{\Delta t} = \frac{{E_{T} }}{\Delta t} + \frac{{E_{C} }}{\Delta t} = S\rho \Delta T(x,y,z,t) + \frac{k\Delta t}{{\Delta x\Delta y\Delta z}}\sum\limits_{i = 1}^{6} {\frac{{A_{i} \Delta T_{i} }}{{l_{i} }}} $$

Now reads:

$$ P = \frac{{\varDelta Q_{H} }}{\varDelta t} = \frac{{E_{T} }}{\varDelta t} + \frac{{E_{C} }}{\varDelta t} = \frac{S\rho \varDelta T(x,y,z,t)}{{\varDelta t}} + \frac{k}{\varDelta x\varDelta y\varDelta z}\sum\limits_{i = 1}^{6} {\frac{{A_{i} \varDelta T_{i} }}{{l_{i} }}} $$

The original Article has been corrected.

